# An unresectable desmoid tumour demonstrating long-term complete response to imatinib monotherapy: a case report

**DOI:** 10.1186/s13256-026-06309-z

**Published:** 2026-06-27

**Authors:** Po Liu, Yat-Ming Lau, Michail Charakidis, Teesha Downton, Yoni Byron, Narayan Karanth

**Affiliations:** 1 Alan Walker Cancer Care Centre, Royal Darwin Hospital, Darwin, NT Australia; 2 School of Medicine, Adelaide University, Adelaide, SA Australia; 3 Faculty of Medicine, Charles Darwin University, Darwin, NT Australia; 4 Faculty of Medicine and Health, Flinders University, Darwin, NT Australia

**Keywords:** Desmoid, Fibromatosis, Imatinib, Unresectable, Mesenteric

## Abstract

**Introduction:**

Desmoid tumours are rare locally invasive soft tissue neoplasms arising from mesenchymal tissue that lack metastatic potential but have a high propensity for local recurrence. For unresectable mesenteric desmoid tumours requiring systemic therapy, the therapeutic landscape spans anti-hormonal combinations, chemotherapy, tyrosine kinase inhibitors, and gamma-secretase inhibitors. Imatinib is a multi-target tyrosine kinase inhibitor targeting PDGFR-alpha/beta and c-KIT that has demonstrated clinical activity in desmoid tumours with a favourable toxicity profile.

**Case presentation:**

We present a case of a 27-year-old white Australian man who presented with a large abdominal mass and weight loss. Imaging revealed a 23 × 12 × 21 cm mass that was diagnosed as a desmoid tumour via biopsy. The tumour was deemed to be unresectable and radiotherapy was considered to carry an unacceptable risk of toxicity. Initial treatment with tamoxifen and sulindac was unable to halt disease progression. The patient was subsequently commenced on imatinib 400 mg daily. Imaging after four months showed partial response, and over six years, complete radiological remission was achieved despite intermittent medication compliance. Aside from one episode of grade 1 photosensitivity, no significant toxicities were observed. Imatinib was self-ceased after ten years of treatment, and the patient remains disease-free at eleven years post-diagnosis under active surveillance.

**Conclusion:**

We describe a case of complete and durable remission achieved with imatinib monotherapy in an unresectable mesenteric desmoid tumour with the patient remaining disease-free at 10 years. This case highlights the potential for long-term complete response and underscores the need for prospective data to guide treatment duration and cessation criteria.

## Introduction

Desmoid tumours (aggressive fibromatoses) are rare, locally invasive soft tissue neoplasms that arise from mesenchymal tissue. Although histologically benign and lacking metastatic potential, they are characterized by an unpredictable clinical course with a high propensity for local recurrence. These tumours account for less than 3% of all soft tissue tumours [1], with an estimated incidence of less than ten cases per million annually [2]. Desmoid tumours are more common in young adults with a median age of diagnosis in the third decade of life [3]. Management strategies should be discussed at a multidisciplinary meeting and are generally tailored to the tumour location, size, growth behaviour, and patient symptoms. While surgical resection was historically considered the primary treatment modality for desmoid tumours, the management paradigm has shifted considerably over the past decade. Active surveillance is now recommended as the frontline approach for all patients with desmoid tumours prior to any active treatment, reserving surgery for specific scenarios such as complications, threats to quality of life, or continuous progression [4]. Radiation, local ablative, and vascular therapies can be considered in select cases for desmoid tumours involving the abdominal wall, extremities, girdles, chest wall, thorax, or head and neck [5]. For mesenteric desmoid tumours requiring treatment, real-world management has transitioned towards systemic therapies [6]. Medical management spans an evolving therapeutic landscape that ranges from anti-hormonal combinations such as tamoxifen with or without non-steroidal anti-inflammatory drugs, to targeted tyrosine kinase inhibitors like imatinib and sorafenib, culminating in the recent approval of the gamma-secretase inhibitor nirogacestat.

Imatinib is a tyrosine kinase inhibitor targeting PDGFR-alpha/beta and c-KIT that has demonstrated clinical activity in select cases of unresectable or progressive desmoid tumours. Imatinib offers a targeted approach with a favourable toxicity profile compared to chemotherapy. Evidence for its use in unresectable desmoid tumours was established by two landmark, prospective, phase II studies [7, 8]. We present a case of complete and durable radiological remission achieved with imatinib monotherapy in a patient with an unresectable mesenteric desmoid tumour, contributing to the growing body of evidence supporting its role in the medical management of this condition.

## Case report/case presentation

A 27-year-old white Australian man presented in 2014 with an abdominal mass that was progressively increasing in size and weight loss of 5 kg over a period of three months. He had a past medical history of Gilbert’s syndrome, paranoid schizophrenia, and prior excessive alcohol use, none of which required pharmacological treatment at the time of presentation. His family history did not reveal any significant malignancy history or predisposition including familial adenomatous polyposis.

Baseline CT imaging showed a large solid vascular mass in the abdomen arising from the mesentery with dimensions of 23 × 12 × 21 cm. Core biopsy of the mass revealed morphological features and an immunohistochemical profile (strong diffuse positivity for SMA) compatible with a diagnosis of mesenteric fibromatosis (desmoid tumour). Staining for c-KIT (CD117) was negative and aberrant faint expression of bcl-2 was noted. Mutational analysis for *CTNNB1* was not performed. Colonoscopy showed moderate haemorrhoids with no concerning lesions or polyps present.

The patient’s case was discussed at a multi-disciplinary meeting which deemed his tumour unresectable due to the high vascularity and size of the lesion. Radiotherapy was considered to have an unacceptable risk of toxicity due to the large tumour size.

**Fig. 1:**
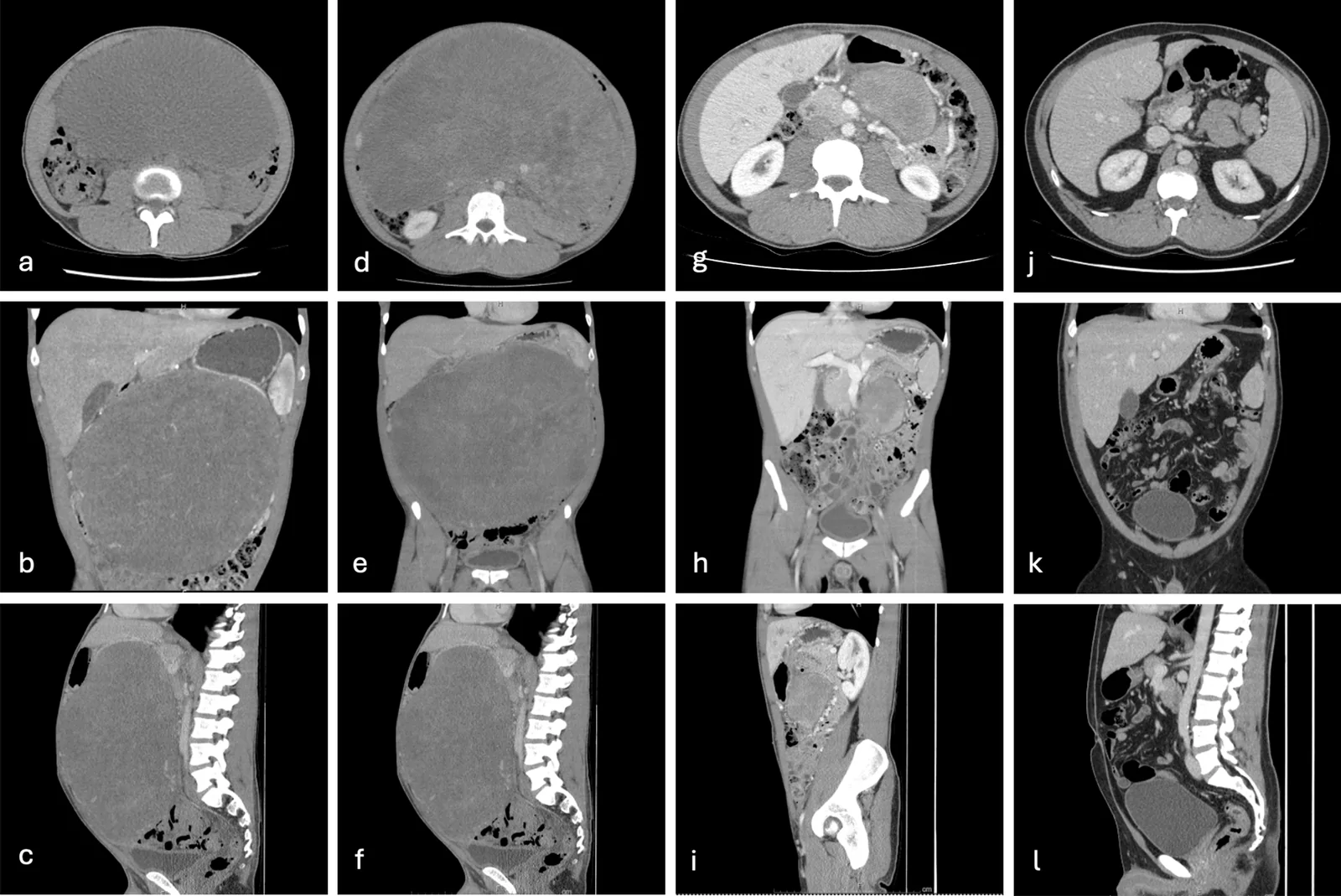
Computed tomography images of mesenteric desmoid tumour during course of treatment. Respective transverse, coronal, sagittal views at diagnosis (**a**–**c**), week 25 after combination tamoxifen and suldinac therapy (**d**–**f**), week 58 while on second-line imatinib (**g**–**i**), and 10.5 years (**j**–**l**)

Systemic therapy was started with tamoxifen 40 mg once daily combined with sulindac 100 mg twice daily. After three months of therapy, the patient was symptomatically better but unable to tolerate sulindac due to reflux. He experienced a further 2 kg of weight loss and had developed bilateral flank pain. CT imaging demonstrated progressive disease with the mass now measuring 30 × 17 × 30 cm with extensive vascularity and mass effect on bowel loops. Sulindac and tamoxifen were ceased, and he was then commenced on second-line imatinib 400 mg once daily. A chronological series of CT imaging can be seen in Fig. 1.

Radiographic reassessment four months later showed partial response with tumour dimensions shrinking to 24 × 11 x 23 cm. The patient’s symptoms had resolved at this stage. Medication compliance with imatinib was intermittent due to the patient’s social and psychiatric factors. Despite intermittent dosing, a continued response to imatinib was observed until imaging in the sixth year of treatment which demonstrated a complete response. Chronological changes in tumour dimensions over the duration of therapy are available in Table 1. No oedema, nausea, or gastrointestinal side effects were reported. The only toxicity identified during therapy was a single episode of grade 1 photosensitivity involving the face. Regular blood tests did not reveal any liver or myelosuppressive toxicity. At approximately ten years from diagnosis, the patient self-ceased imatinib given the perceived durable response and lack of further benefit of continuing indefinite therapy. He remains symptom-free at the time of this writing and is currently under active surveillance at eleven years post-diagnosis.

**Table 1.** Measurements on serial computed tomography imaging in transverse and sagittal planes of patient with mesenteric desmoid tumour

Time from diagnosis (weeks)	Transverse dimensions (mm)	Sagittal length (mm)
0	230 × 120	210
25	303 × 170	297
42	116 × 244	237
58	87 × 52	85
72	49 × 45	29
87	38 × 22	–
106	31 × 17	–
122	19 × 13	39
152	Complete response	–

Dashes (−) indicate size not formally reported

## Discussion

Systemic therapy options for unresectable desmoid tumours have included non-steroidal anti-inflammatory drugs (NSAIDs); hormonal agents such as tamoxifen; chemotherapy such as methotrexate in combination with vinorelbine or vinblastine, and liposomal doxorubicin; tyrosine kinase inhibitors including imatinib and sorafenib; and nirogacestat [5]. Phase III trial data exist only for nirogacestat [9] and sorafenib [10], and the National Comprehensive Cancer Network (NCCN) guidelines reflect this evidence with category 1 endorsement for their use [5]. The optimal choice and sequencing of therapy is currently unknown given the low incidence of this disease. Imatinib was chosen in our case as a second-line agent primarily because of a lack of access to alternative options and the data for nirogacestat and sorafenib were not yet available at the time of disease progression.

Imatinib has been shown to have activity against desmoid tumours in several phase II studies [7, 8, 11–13]. The French Sarcoma Group (FNLCC) performed a trial involving 35 patients with desmoid tumours given 400 mg once daily of imatinib and observed three partial responses and one complete response assessed by blinded independent committee [7]. The study reported a median progression-free survival of 25 months and grade 3 toxicities were observed in 45% of patients. The Sarcoma Alliance for Research through Collaboration (SARC) group conducted the largest multi-centre trial consisting of 51 patients, with imatinib dosing based on body surface area rather than a flat dosing regimen used in our case [8]. Patients with BSA > 1.5 m^2^ received 300 mg twice daily, 1.0 to 1.49 m^2^ received 200 mg twice daily, and BSA of < 1.0 m^2^ received 100 mg twice daily. The study only observed partial responses in three patients at four months of therapy and no complete responders were reported. A further study by Heinrich et al. using 400 mg twice daily dosing of imatinib found that three out of 19 patients had a partial response lasting more than 1.5 years and only a single patient showed an objective response of greater than 3 years [11]. The German Interdisciplinary Sarcoma Group (GISG) study recruited 38 patients and gave a daily imatinib dose of 800 mg over 2 years which could be continued on nilotinib if found to have disease progression [12]. The study described seven partial responses after 21 months of follow-up. Real-world data from a large retrospective study of 200 patients with desmoid tumour across two tertiary centres in India found that across all treatment lines, imatinib produced stable disease in 52.1% and partial response in 41.3% of patients [6]. Notably, no complete responses were documented in that cohort, further highlighting the exceptional nature of the complete and durable response in our patient.

There is limited high-quality data to guide the precise duration of tyrosine kinase inhibitor therapy. FNLCC and GISG trials gave imatinib for a set period of one and two years respectively [7, 12], whilst both the SARC trial and Heinrich et al.’s study treated continuously until progression, toxicity, or withdrawal [8, 11]. Consensus opinion from the international Desmoid Tumour Working Group recommends a minimum of 6 months of therapy prior to assessing for efficacy but does not offer a specified time to withdraw therapy [4]. Our patient continued imatinib monotherapy until he self-ceased at 10 years with complete radiological response observed at six years. Further data from large prospective randomized controlled trials would provide valuable insights to guide clinical decision making on cessation of therapy but these data are currently lacking.

Imatinib’s mechanism of action in the management of desmoid tumours has been hypothesized to involve multi-target inhibition of c-KIT, ABL, ARG, and PDGFR-alpha/beta tyrosine kinases [11]. Studies attempting to examine this hypothesis have shown PDGFR-alpha [14] and PDGFR-beta [11] expression in a large proportion of tumours although differences in staining assays do present a challenge in making cross study comparisons. An attempt to correlate immunohistochemistry findings with clinical outcomes was unfortunately unsuccessful [8]. While c-KIT was negative on staining and we did not test for PDGFR-alpha/beta in our case, we speculate that genomic alterations in one or multiple of the imatinib targets potentially provided the molecular susceptibility to long-term imatinib monotherapy. Counter-intuitively, we noted that faint expression of bcl-2 was present on initial immunohistochemical staining in our patient’s tumour of which upregulation has been shown in imatinib resistance in chronic myeloid leukaemia [15]. The clinical significance of this finding and how it relates to the outcomes we observed is highly speculative and requires further exploration. A recent Korean multi-centre phase II trial conducted whole-genome sequencing on desmoid tumours prior to treatment with imatinib to identify potential biomarkers [13]. The study found that *HES1* overexpression, a downstream target of *NOTCH2* signaling, was significant in discriminating a patient’s responsiveness to treatment. The results raise a separate hypothesis that underlying differences in Notch pathway activity may modulate response to imatinib.

An alternative explanation for the observed outcomes would be the spontaneous regression of the tumour that can be observed in up to 20% of cases [16]. However, we believe this to be unlikely in our case as the tumour demonstrated growth despite three months of combination tamoxifen and NSAID therapy and regression was only observed after commencement of imatinib monotherapy. In addition, no recurrence was found in follow-up after 10 years of therapy suggesting that imatinib played a significant role in the regression.

## Conclusion

We report the ten-year follow-up of a massive unresectable desmoid tumour of the mesentery achieving complete radiological remission on imatinib monotherapy. This case demonstrates that durable complete response is achievable with imatinib, even in the context of intermittent medication compliance, and that treatment cessation after sustained remission may be feasible. These findings support the continued consideration of imatinib in the systemic management of unresectable desmoid tumours and highlight the need for further prospective data to guide optimal treatment duration and cessation criteria.

## Data Availability

The data that support the finding of this study are provided in the published article. Requests for additional information can be directed to the corresponding author.
